# No differences in outcomes with stopping or continuing antibiotic suppression in periprosthetic joint infections

**DOI:** 10.5194/jbji-9-143-2024

**Published:** 2024-05-14

**Authors:** Daisuke Furukawa, Megan Dunning, Sa Shen, Amy Chang, Jenny Aronson, Derek F. Amanatullah, Gina A. Suh, Shanthi Kappagoda

**Affiliations:** 1 Division of Infectious Diseases and Geographic Medicine, Stanford University, Stanford, California 94304, USA; 2 Quantitative Sciences Unit, Stanford University, Stanford, California 94304, USA; 3 Department of Orthopaedic Surgery, Stanford University, Redwood City, California 94063, USA; 4 Division of Public Health, Infectious Diseases, and Occupational Medicine, Mayo Clinic, Rochester, Minnesota 55902, USA

## Abstract

The data on long-term antibiotic use following debridement, antibiotics, and implant retention (DAIR) for treatment of periprosthetic joint infections are limited. In this single-center retrospective study, we show that patients with eventual cessation of antibiotic suppression after DAIR had similar outcomes to those who remained on chronic antibiotic suppression.

## 1 Introduction

Debridement, antibiotics, and implant retention (DAIR) is a surgical approach for treatment of periprosthetic joint infections (PJI) that is favored in certain patients such as those with short symptom duration, good soft tissue, and stable implants (Beam and Osmon, 2018; Osmon et al., 2013). The ideal antibiotic duration after DAIR is unclear. The 2012 Infectious Diseases Society of America (IDSA) PJI guideline states that indefinite oral antimicrobial suppression may follow standard treatment course and that the guideline panel could not agree on the use and duration of chronic antibiotic suppression (Osmon et al., 2013). The body of evidence is limited with a few retrospective studies suggesting potential benefit with antibiotic suppression (Byren et al., 2009; Bryan et al., 2017; Renz et al., 2019; Siqueira et al., 2015). However, as discussed in a review by Cortes-Penfield et al. (2024), these studies are highly heterogenous with regards to duration of suppression, definition of outcomes, and patient population, and it still remains unclear if and for whom antibiotic suppression is beneficial. In this study, we aimed to describe the failure rates in patients treated with DAIR and evaluate the role of long-term antibiotic suppression. We hypothesized that long-term antibiotic suppression would lower rates of failure.

## 2 Materials and methods

We conducted a single-center retrospective study of patients who underwent DAIR for PJI. Patients with International Classification of Diseases 9th and 10th revision codes for PJI (996.6, T84.5, T84.6, T84.7) who were admitted and seen in our orthopedic-infectious diseases clinic from 1 January 2013 to 31 December 2019 were queried from our institution's clinical data warehouse. Patients with knee or hip PJIs who underwent DAIR were then identified through manual chart review. Exclusion criteria included PJI of other joints, tumor prosthesis, not meeting Musculoskeletal Infection Society (MSIS) criteria for PJI (Parvizi et al., 2018), and being lost to follow-up prior to completion of intravenous (IV) therapy. Patient characteristics, antibiotic type and duration, and laboratory values were abstracted through manual chart review and entered into REDCap. Treatment failure was defined as repeat surgery for clinical suspicion of infection leading to repeat treatment with a prolonged course of antibiotics recommended by the consulting infectious diseases provider. Antibiotic suppression was defined as oral antibiotics given after completion of IV therapy. Our standard practice at the time of this chart review was to treat patients with 6 weeks of IV therapy prior to transitioning to oral antibiotics. Antibiotics were selected based on IDSA guidelines, and rifampin was considered for patients with staphylococcal infections at the dose and duration recommended by the guideline (Osmon et al., 2013).

Our exploratory analysis identified that patients who did not meet guideline-recommended criteria for DAIR were included in our cohort. To account for this heterogeneity in patient cohort, we stratified our analysis based on whether the patient met criteria for DAIR as defined by the 2012 IDSA PJI guideline (well-fixed prosthesis without a sinus tract and within 30 d of prosthesis implantation or within 3 weeks of symptom onset) (Osmon et al., 2013).

Results were analyzed descriptively. We used the chi-square test and Fisher's exact test for categorical variables and the Wilcoxon rank-sum test for continuous variables for group comparisons. We used STATA version 16 software (STATA Corp, College Station, TX) for all analyses.

## 3 Results

A total of 96 patients were included in our cohort of whom 68 (71 %) met criteria for DAIR. Of the 28 (29 %) patients who did not meet criteria for DAIR, the reason for not meeting criteria was prolonged symptom duration ( n=25 ) and presence of sinus tract ( n=3 ). The documented reasoning for pursuing DAIR despite not meeting criteria was that two-stage exchange was considered technically not feasible or challenging ( n=9 ), infection was not suspected peri-operatively ( n=5 ), patient preference ( n=3 ), and unable to abstract from chart review ( n=11 ).

**Table 1:** Baseline patient characteristics and comparison of patients grouped by treatment outcomes and status of oral suppression.

	Total	Success	Fail	p value	Discontinued oral	Continued on oral	p value
	N (%)/	N (%)/	N (%)/		suppression N (%)/	suppression N (%)/	
	median (IQR)	median (IQR)	median (IQR)		median (IQR)	median (IQR)	
Total	96	80	16		55	35	
Age (years)	67.2 (59.2–75.3)	66.1 (57.7–75.9)	67.2 (60.1–74.3)	0.62	68.4 (63.6–76.0)	63.1 (57.1–73.8)	0.11
Male	59 (62)	49 (61)	10 (63)	1	32 (58)	23 (66)	0.51
Body mass index (kg m^-2^)	29.1 (24.6–34.2)	28.5 (24.4–33.7)	33.2 (26.9–39.3)	0.09	29.0 (24.5–34.2)	29.8 (25.6–34.0)	0.79
Joint				0.17			1
Hip	58 (60)	51 (64)	7 (44)		33 (60)	25 (61)	
Knee	38 (40)	29 (37)	9 (57)		22 (40)	16 (39)	
Days of symptoms	8 (4, 24)	8 (4–24)	6.5 (4.5–31.5)	0.98	9 (5–43)	7 (3–15)	0.16
Met criteria for DAIR	68 (71)	56 (70)	12 (75)	0.69	39 (71)	24 (69)	0.82
Bacteremia	15 (16)	10 (13)	5 (31)	0.12	9 (16)	6 (15)	1
Early post-operative infection	34 (35)	29 (36)	5 (31)	0.78	19 (35)	12 (34)	1
Acute hematogenous infection	30 (31)	23 (29)	7 (44)	0.25	16 (29)	12 (34)	0.65
Past medical history							
Autoimmune disease	13 (14)	13 (16)	0 (0)	0.12	6 (11)	7 (20)	0.36
Diabetes mellitus	17 (18)	13 (16)	4 (25)	0.47	10 (18)	6 (17)	1
Smoking	5 (5)	3 (4)	2 (13)	0.19	1 (2)	3 (9)	0.3
End-stage renal disease	2 (2)	2 (3)	0 (0)	1	0 (0)	2 (6)	0.15
Cirrhosis	2 (2)	2 (3)	0 (0)	1	1 (2)	1 (3)	1
Coronary artery disease/congestive heart failure	26 (27)	25 (31)	1 (6)	0.06	16 (29)	10 (29)	1
Malignancy	9 (9)	8 (10)	1 (6)	1	7 (12)	1 (3)	0.14
Immunosuppressed	9 (9)	8 (10)	1 (6)	1	3 (5.5)	5 (14.3)	0.25
History of PJI same joint	27 (28)	22 (28)	5 (31)	0.77	13 (24)	12 (34)	0.34
History of PJI different joint	5 (5)	4 (5)	1 (6)	1	3 (5)	2 (6)	1
C-reactive protein (mg dL^-1^)	9.1 (3–19.9)	7.6 (3–16)	21.6 (11.9–31.6)	0.01	7.4 (2.5–21.4)	9.3 (3.5–16.7)	0.74
Erythrocyte sedimentation rate (mm h^-1^)	60.5 (36.0–85.0)	56 (32–84)	64.5 (58–97.5)	0.33	60 (36–85)	54 (29–84)	0.77
Synovial nucleated cell count (cells µ L^-1^)	43 581 (13 212–81 399)	43 581 (11 401–77 496)	53 311 (24 827–87 964)	0.34	34 839 (3618–68 375)	46 977 (17 189–91 926)	0.22
Synovial neutrophils (%)	94 (87–97)	94 (87–97)	93 (92–96)	0.788	91 (84–95)	97 (95–98)	< 0.01
C-reactive protein end of IV (mg dL^-1^)	1.0 (0.3–3.1)	1.0 (0.3–2.5)	1.0 (0.7–3.1)	0.38	1 (0.3–3.1)	1.0 (0.5–2.2)	0.88
Erythrocyte sedimentation rate end of IV (mm h^-1^)	31 (16.0–44)	29.5 (15–40)	31.5 (22–85)	0.2	28 (15–36)	35 (17–50)	0.2
Microorganism							
*Staphylococcus aureus*	25 (26)	19 (24)	6 (38)	0.35	14 (25)	7 (20)	0.62
Coagulase negative *Staphylococcus*	25 (26)	22 (28)	3 (19)	0.55	15 (27)	10 (29)	1
Other gram-positive coccus^a^	17 (18)	15 (19)	2 (13)	0.73	9 (16)	8 (23)	0.58
Gram-negative rods^b^	14 (15)	10 (13)	4 (25)	0.24	7 (13)	5 (14)	1
Other^c^	12 (13)	10 (13)	2 (13)	1	8 (14.6)	3 (9)	0.52
Polymicrobial	8 (8)	6 (8)	2 (13)	0.62	5 (9)	2 (6)	0.7
Initial antibiotic therapy							
IV penicillin	10 (10)	9 (11)	1 (6)	1	5 (9)	5 (14)	0.5
IV cephalosporin	48 (50)	38 (48)	10 (66)	0.41	25 (45)	18 (51)	0.67
Carbapenem	9 (9)	8 (10)	1 (6)	1	7 (12)	1 (3)	0.14
Vancomycin	28 (29)	24 (30)	4 (25)	0.77	16 (29)	12 (34)	0.65
Daptomycin	6 (6)	5 (6)	1 (6)	1	3 (5)	3 (9)	0.67
Rifampin	46 (48)	39 (49)	7 (44)	0.79	28 (51)	15 (43)	0.52

**Table 1:** Continued.

	Total	Success	Fail	p value	Discontinued oral	Continued on oral	p value
	N (%)/	N (%)/	N (%)/		suppression N (%)/	suppression N (%)/	
	median (IQR)	median (IQR)	median (IQR)		median (IQR)	median (IQR)	
Duration of IV (days)^d^	46 (42–56)	46 (42–56.5)	44.5 (33–53)	0.24	45 (42–53)	48 (42–66)	0.14
Oral antibiotic suppression^d^		80	10				
Penicillin	30 (33)	26 (33)	4 (40)	0.73	18 (33)	12 (34)	1
Cephalosporin	20 (22)	18 (23)	2 (20)	1	12 (22)	8 (23)	1
Trimethoprim-sulfamethoxazole	19 (21)	17 (21)	2 (20)	1	10 (18)	9 (26)	0.43
Linezolid	2 (2)	2 (3)	0 (0)	1	2 (4)	0 (0)	0.52
Doxycycline	35 (39)	32 (40)	3 (30)	0.74	20 (36)	15 (43)	0.66
Fluoroquinolone	13 (14)	11 (14)	2 (20)	0.63	11 (20)	2 (5.7)	0.07
Discontinued oral suppression^d^	55 (61)	49 (61)	6 (60)	1	n/a	n/a	n/a
Duration of oral suppression (days)^e^	184 (120–576)	189 (133–576)	163 (63–348)	0.39	184 (120–576)	798 (210–1458)	< 0.01
Days to failure or final follow-up	816 (313.5–1279)	869.5 (414–1470)	154 (33–545)	< 0.01	853 (396–1340)	850 (279–1513)	0.66
Treatment failure	16 (17)	n/a	n/a	n/a	6 (11)	4 (11)	1

^a^ *Streptococcus* spp. ( n=13 ), *Enterococcus* spp. ( n=4 ). ^b^ *Enterobacterales* ( n=13 ), *Pseudomonas* *aeruginosa* ( n=1 ). ^c^ *Cutibacterium acnes* (n = 3), *Corynebacterium* spp. ( n=2 ), *Bacillus* spp. ( n=1 ), *Burkholderia* *cenocepacia* ( n=1 ), *Campylobacter fetus* ( n=1 ), *Haemophilus parainfluenzae* ( n=1 ), *Stenotrophomonas maltophilia* ( n=1 ), other anaerobes ( n=3 ). ^d^ Analysis restricted to those that completed IV therapy ( n=90 ). ^e^ Analysis restricted to those that discontinued suppression ( n=55 ). n/a – not applicable

In the entire cohort, treatment failure was seen in 16 patients (17 %), of which 6 failed while on IV therapy and 12 failed within the first year. Median time to failure was 154 (IQR 33–545) days. Of 16 failures, 6 were due to the same organism as the index infection, and 4 were due to a different organism. The remaining 6 had negative cultures at time of repeat surgery, but all were on antibiotics when clinical failure was suspected. Rifampin was used in 46 (48 %) patients, with a median duration of 89 (IQR 58–145) days. Pre-operative C-reactive protein (CRP) levels were higher in the failure group compared to the success group (21.6 vs 7.6 mg dL^-1^, p=0.01 ), and there was no significant difference in the proportion of patients meeting criteria for DAIR (70 % success group vs 75 % failure group; p=0.69 ) or the proportion of patients remaining on suppressive antibiotic therapy (39 % success group vs 40 % failure group; p=1.0 ) (Table 1).

**Figure 1:**
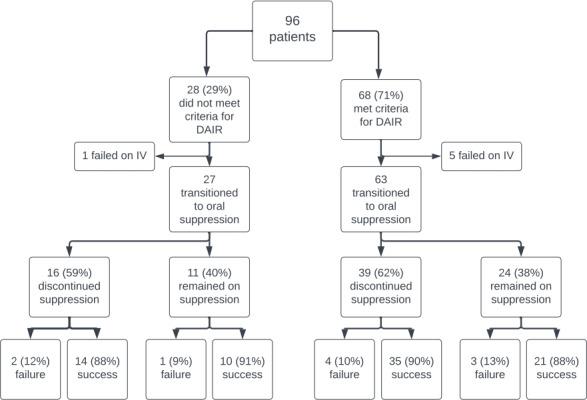
Flowchart of patient outcomes.

In the subset of patients who were transitioned to oral antibiotics, those who discontinued oral suppression had the same failure rates as those who were continued on oral suppression (11 % vs 11 %, p=1.0 ) (Table 1). Between the two groups, synovial percent neutrophil was the only factor that was significantly different (91 % [IQR 85–95] for the group that discontinued suppression vs 97 % [IQR 95–98] for the group that continued suppression; p<0.01 ), and there was no difference in the proportion of patients meeting criteria for DAIR (71 % vs 69 %; p=0.82 ) or median duration of follow-up (853 d vs 850 d; p=0.66 ). Incidence of treatment failure was similar regardless of the status of oral suppression, and this held true even after stratifying the analysis by meeting or not meeting DAIR criteria (Fig. 1). Patient data comparing those who did and did not meet criteria for DAIR are presented in Table A1 of the Appendix.

## 4 Discussion

Overall, our study suggests that DAIR is an acceptable treatment strategy for patients with knee or hip PJIs, and our outcomes are even more reassuring as the majority of patients eventually stopped antibiotic therapy, sparing them from ongoing risk of adverse events Importantly, we demonstrated that outcomes were similar for patients who remained on suppressive antibiotics compared to those who discontinued suppression.

Other studies have demonstrated diminishing benefit with long term antibiotic use, arguing against indefinite suppressive antibiotics. Shah et al. (2020) showed that the benefit of suppression plateaued after 1 year of suppression, and Tai et al. (2022) similarly showed no differences in risk reduction between 1 year and 5 years of suppression. On closer observation of our data, three out of six patients who failed off suppression experienced failure either by a different organism or after over 2 years had passed since cessation of antibiotics. This may suggest that these failures were due to de novo acquisition of a new infection, rather than recurrence, thus overestimating rates of treatment failure. Though an argument could be made that being on suppressive antibiotics may have prevented new infections, the role of prophylactic antibiotics is not well established in this patient population and would not be a justification for indefinite suppression.

Interestingly, in our cohort, patients not meeting criteria for DAIR had similar overall failure rates to those who met criteria for DAIR, and the failure rates were similar even after stratifying the cohort by the status of chronic suppressive antibiotics. Though we would not suggest that DAIR should be considered for all patients regardless of timing of infection or symptom duration, our study does suggest that for some patients, outcomes may still be favorable even for those who do not meet guideline-recommended criteria for DAIR.

Our study has a number of limitations. First, this was a single-center retrospective study, and thus generalizability may be limited. Additionally, given the retrospective nature, there may have been bias unaccounted for in our analysis. Lastly, given the small sample size, our main findings were largely descriptive, and a more robust statistical analysis was not possible, which limited our ability to account for all confounders. We did however attempt to explore potential confounders by presenting data on patient characteristics based on DAIR criteria and antibiotic suppression. Notably, the major significant differences that we observed were that patients who met indication for DAIR had indices suggestive of acute inflammatory state (shorter symptom duration, higher CRP, higher synovial nucleated cells) (Table A1), which are expected differences considering the DAIR criteria. Regardless, based on our descriptive statistics alone, our study indicates that patients with eventual cessation of oral suppression still had high rates of success.

## 5 Conclusions

In our retrospective cohort study, patients with eventual cessation of antibiotics after DAIR had comparable outcomes to those who remained on indefinite oral antibiotic suppression. 

## Data Availability

Data used and analyzed are available upon reasonable request.

## Appendix A

**Table A1:** Comparison of patients meeting DAIR criteria versus patients not meeting DAIR criteria.

	Meeting DAIR criteria	Not meeting DAIR criteria	
	N (%)/median (IQR)	N (%)/median (IQR)	p value
Total	68	28	
Age (years)	66.6 (58–73.7)	71.1 (61.2–77.0)	0.33
Male	43 (63)	16 (57)	0.65
Body mass index (kg m^-2^)	29.8 (26.4–34.3)	27.2 (23.8–32.5)	0.13
Joint			0.37
Hip	39 (58)	19 (69)	
Knee	29 (43)	9 (32)	
Days of symptoms	5.5 (3–9)	68 (29.5–161)	< 0.01
Bacteremia	15 (22)	0 (0)	0.005
Past medical history			
Autoimmune disease	7 (10)	6 (21)	0.19
Diabetes mellitus	13 (19)	4 (14)	0.77
Smoking	4 (6)	1 (4)	1
End-stage renal disease	0 (0)	2 (7)	0.08
Cirrhosis	2 (3)	0 (0)	1
Coronary artery disease/congestive heart failure	21 (31)	5 (18)	0.22
Malignancy	7 (10)	2 (7)	1
Immunosuppressed	7 (10)	2 (7)	1
History of PJI same joint	16 (24)	11 (39)	0.14
History of PJI different joint	4 (6)	1 (4)	1
C-reactive protein (mg dL^-1^)	12.8 (4.8–26.1)	6 (1.9–10.4)	< 0.01
Erythrocyte sedimentation rate (mm h^-1^)	62.5 (37–94)	53.5 (36–78)	0.36
Synovial nucleated cell count (cells µ L^-1^)	56 840 (20 907.1–113 370)	13 211 (709.6–39 923.2)	< 0.01
Synovial neutrophils (%)	93 (87–97)	95.5 (84–97)	0.73
C-reactive protein end of IV (mg dL^-1^)	1.1 (0.47–3.1)	0.69 (0.3–1.8)	0.21
Erythrocyte sedimentation rate end of IV (mm h^-1^)	29 (16–45)	32 (13–38)	0.80
Microorganisms			
*Staphylococcus aureus*	22 (32)	3 (11)	0.04
Coagulase negative *Staphylococcus*	16 (24)	9 (32)	0.45
Other gram-positive coccus	13 (19)	4 (14)	0.77
Gram-negative rods	9 (13)	5 (18)	0.54
Other	8 (12)	4 (14)	0.74
Polymicrobial	7 (10)	1 (4)	0.43
Initial antibiotic therapy			
IV penicillin	7 (10)	3 (1)	1
IV cephalosporin	34 (50)	14 (50)	1
Carbapenem	6 (9)	3 (11)	0.72
Vancomycin	20 (29)	8 (29)	1
Daptomycin	4 (6)	2 (7)	1
Rifampin	35 (51)	11 (39)	0.37
Duration IV (days)^a^	46 (42–57)	46 (43–56)	0.89
Oral antibiotic suppression^a^	63	27	
Penicillin	24 (38)	6 (22)	0.22
Cephalosporin	14 (22)	6 (22)	1
Trimethoprim-sulfamethoxazole	13 (21)	6 (22)	1
Linezolid	2 (3)	0 (0)	0.54
Doxycycline	24 (38)	11 (41)	0.82
Fluoroquinolone	9 (14)	4 (15)	1
Discontinued oral suppression^a^	39 (62)	16 (59)	0.82
Duration of oral antibiotics (days)^b^	164 (87–489)	294.5 (133.5–670.5)	0.39
Treatment failure	12 (18)	4 (14)	0.77
Days to failure or final follow-up	746.5 (278.5–1106.5)	1140 (627–1674.5)	0.01

^a^ Analysis restricted to those that completed IV therapy ( n=90 ). ^b^ Analysis restricted to those that discontinued suppression ( n=55 ).
